# Usefulness of central venous saturation as a predictor of thiamine deficiency in critically ill patients: a case report

**DOI:** 10.1186/s40560-017-0255-7

**Published:** 2017-11-06

**Authors:** Genri Numata, Satoshi Kodera, Hiroyuki Kiriyama, Atsuko Nakayama, Eisuke Amiya, Arihiro Kiyosue, Masaru Hatano, Eiki Takimoto, Masafumi Watanabe, Issei Komuro

**Affiliations:** Department of Cardiovascular Medicine, Graduate school of Medicine, The University of Tokyo, 7-3-1 Hongo, Bunkyo-ku, Tokyo, 1130033 Japan

**Keywords:** Thiamine, Oxygen consumption, Beriberi, Shock, Diuretics, Central venous catheter

## Abstract

**Background:**

Central venous oxygen saturation (ScvO_2_) reflects the balance of oxygen delivery and consumption. Low ScvO_2_ indicates the presence of inadequate oxygen delivery, while high ScvO_2_ indicates reduced oxygen consumption and is sometimes associated with a high mortality rate in critically ill patients from dysoxia. Thiamine is an essential cofactor in cellular aerobic metabolism. Thiamine deficiency is more prevalent than was previously thought, and underlies severe conditions in critically ill patients. However, currently, there is no rapid diagnostic test for thiamine deficiency. Considering oxygen flux, high ScvO_2_ might be associated with thiamine deficiency.

**Case presentation:**

A 70-year-old man admitted to the hospital with chief complaint of malaise and edema. He was diagnosed with heart failure with preserved ejection function and was treated with loop diuretics, which resulted in shock. Venoarterial extracorporeal membrane oxygenation and intra-aortic balloon pumping was indicated. The right heart catheter showed high ScvO_2_, normal cardiac output, and low systemic vascular resistance. Thiamine deficiency was suspected and we started the thiamine infusion. His hemodynamic status improved after thiamine replacement. After his recovery, it was discovered that he had a 1-month history of anorexia and thiamine deficiency. His final diagnosis was beriberi.

**Conclusions:**

The current case showed the relation between thiamine deficiency and high ScvO_2_. A literature review also suggested that thiamine deficiency is associated with high ScvO_2_. Thiamine deficiency causes impaired tissue oxygen extraction, which could lead to high ScvO_2_. In this context, high ScvO_2_ might serve as a predictor of thiamine deficiency in critically ill patients.

## Background

Central venous oxygen saturation (ScvO_2_) provides an estimate of oxygen flux, reflecting balance of oxygen delivery and consumption. Low ScvO_2_ indicates the presence of inadequate oxygen delivery and ScvO_2_ < 70% has been accepted as a maker for hypo-perfusion in the management of shock patients or postoperative patients. On the other hand, high ScvO_2_ indicates reduced oxygen consumption in systemic organs typically from dysoxia with mitochondrial dysfunction and has been associated with a high mortality rate in critically ill patients [1–3].

Thiamine, a cofactor for pyruvate dehydrogenase (PDH), plays an essential role in cellular aerobic metabolism. Growing evidence suggests that thiamine deficiency is more prevalent than was previously thought, and underlies severe conditions in critically ill patients [3]. However, currently, there is no rapid diagnostic test for thiamine deficiency and thiamine activity, which is useful for diagnosis, is not easily detected in some countries. In the presence of thiamine deficiency, PDH fails to convert pyruvate into acetyl-CoA that enters Krebs cycle to produce adenosine triphosphate (ATP) using oxygen, and thus oxygen consumption is reduced in various tissues. Therefore, high ScvO_2_ values might be a good and convenient marker for thiamine deficiency in critically ill patients. Here, we report a case of heart failure, who was successfully diagnosed with thiamine deficiency using high ScvO_2_ values after presenting shock from diuretics treatment.

## Case presentation

A 70-year-old man presented to our hospital with exaggerated general malaise and leg edema. His blood pressure on admission was 90/50 mmHg, heart rate was 84 beats/min, body temperature 36.5 °C, and respiratory rate 20/min with 99% oxygen saturation on ambient air. He had pitting edema of the lower legs and he gained about 10 kg weight. Blood tests showed mild renal and hepatic impairment (blood urea nitrogen; 39 mg/dL; creatinine, 1.3 mg/dL; aspartate aminotransferase, 52 U/L; alanine transaminase, 53 U/L; gamma-glutamyl transferase, 177 U/L; alkaline phosphatase, 296 U/L), and the brain natriuretic peptide level was 317 pg/mL. There was no evidence of neurological abnormality. A plain radiograph of his chest revealed heart dilation and pleural edema. Transthoracic echocardiography (TTE) showed mild pericardial effusion, an ejection fraction of 56%, mild tricuspid valve regurgitation (right ventricular systolic pressure, 44 mmHg), and the diameter of inferior vena cava of 22 mm with no respiratory variations. He drank 40 to 80 g of alcohol a day. Because he was diagnosed with heart failure with preserved ejection fraction and volume overload, he was treated with infusion of loop diuretics (furosemide 20 mg/day). However diuretics did not work well (urine output, 500 ml/day) and 1 day after starting the diuretic infusion his malaise worsened. His systolic blood pressure suddenly decreased to 50 mmHg. Electrocardiography showed rapid atrial fibrillation (Heart rate, 150 beats/min), low voltage at limb leads, no ST-T change and no prolonged QT interval. TTE showed a little increased pericardial effusion with normal ejection fraction. Cardiac tamponade and atrial fibrillation might lead to his shock status therefore we performed electrical cardioversion and pericardiocentesis, resulting in no response. Fluid resuscitation (1 L) and norepinephrine (1 μg/kg/min) also did not improve his hemodynamic status. Thus we began venoarterial extracorporeal membrane oxygenation (ECMO) and intra-aortic balloon pumping (IABP). Before ECMO and IABP induction, the right heart catheter showed his ScvO_2_ to be 92%, cardiac index 2.2 L/min/m^2^, pulmonary artery wedge pressure 28 mmHg, mean pulmonary artery pressure 36 mmHg, and systemic vascular resistance 596 dyne∙s/cm^5^ on dobutamine 10 μg/kg/min and norepinephrine 1 μg/kg/min. Coronary angiography showed no stenosis and endomyocardial biopsy revealed no significant sings of myocarditis or cardiomyopathy.

**Fig. 1:**
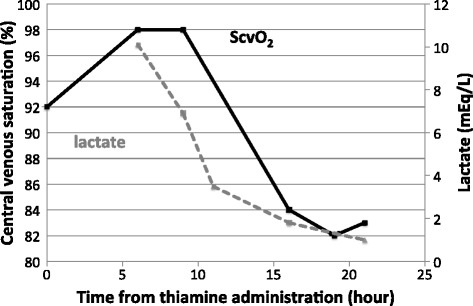
Central venous saturation and serum lactate levels after thiamine administration. Note that both parameters recovered soon after the thiamine replacement therapy

As low systemic vascular resistance and high ScvO_2_ might be caused by thiamine deficiency, we submitted blood sample for thiamine level and started an intravenous infusion of 100 mg thiamine. There was no evidence of infection or adrenal insufficiency. At 24 h after the thiamine administration, his ScvO_2_ decreased from 92 to 82%, and his lactate level decreased, from 90.9 to 9.0 mg/dL (Fig. 1). His blood pressure recovered to the normal range. We thus stopped the catecholamine administration and ECMO. Five days after his recovery from shock, we discovered that his thiamine level at the point of ECMO induction was 14 ng/mL (normal range 24–66 ng/mL). Also, we obtained the additional information on a 1-month history of anorexia. After 2-week thiamine replacement, his malaise and edema disappeared with no other treatments diuretics. His ScvO_2_ before discharge was 60%. Together all, a diagnosis of cardiac beriberi was confirmed.

## Discussion

The current case revealed two important points. High ScvO_2_ might be associated with thiamine deficiency, and a patient’s hemodynamic status in the presence of thiamine deficiency could become worse with the administration of loop diuretics.

Thiamine (i.e., vitamin B1) is a water-soluble vitamin, and thiamine pyrophosphate, the active form of thiamine, serves as a catalyst of PDH in the reactions of pyruvate to acetyl coenzyme A and α-ketoglutarate to succinyl coenzyme A in the Krebs cycle, contributing to the synthesis of ATP. Thus, thiamine deficiency could cause impaired tissue oxygen extraction leading to lactic acidosis with high ScvO_2_. Lactate is actually very helpful for management of shock patients and our data actually showed the parallel transition in ScvO_2_ and lactate. However, lactate alone might be insufficient to evaluate the tissue oxygen flux. Elevated level of lactate is the result of impaired tissue oxygenation and it does not reveal the cause. On the other hand, ScvO_2_ gives us the information about the tissue oxygen consumption and not insufficient oxygen delivery.

In the literature, we found only one report suggesting a relation between cardiac beriberi and high ScvO_2_ (in a case of pediatric heart failure) [4] and no papers exploring the possibility of a relation between high ScvO_2_ and thiamine deficiency in critically ill adults.

In this case, the loop diuretics might have exacerbated the patient’s thiamine deficiency by increasing urinary excretion of thiamine. Although recent studies revealed that the chronic use of loop diuretics might not be associated with thiamine deficiency because of diuretic resistance [5, 6], a short-term use of loop diuretics in combination with other diuretics could induce thiamine deficiency via inhibiting its reabsorption [7].

Some patients with multi-organ failure, such as septic shock, show high ScvO_2_, and it has been implicated as a poor prognostic factor [1]. Although it is conceivable that mitochondrial dysfunction underlies high ScvO_2_ values and has received considerable attention, no consensus has been reached as to its precise pathological mechanism [8]. Thiamine deficiency might play some role in mitochondrial dysfunction in shock patients with high ScvO_2_.

We conducted a literature review using PubMed and Google Scholar. English language case reports published between 1990 and 2017 were reviewed using the search terms” beriberi,”” shock,” and” thiamine.” The review identified five articles describing ScvO_2_ [4, 9–12]. Each case described high ScvO_2_ (Table 1). This finding may support our hypothesis concerning the association of high ScvO_2_ and thiamine deficiency.

**Table 1:** Previously reported cases of high central venous saturation associated with thiamine deficiency and shock

Author (ref)	Year	Patient character	ScvO_2_ (%)	Lactate (mg/dL)	Thiamine concentration (ng/mL)
Misumida et al. [9]	2014	61-year-old man	78	75	11
Majima et al. [4]	2013	1-year-and-4-mo-old boy	82	114	12
Kountchev et al. [10]	2005	50-year-old man	78	186.5	Not examined
Diltoer et al. [11]	2004	62-year-old woman	69	122.5	Below the detection limit
Ito et al. [12]	2002	73-year-old man	87	196.8	16

In a recent randomized trial, patients with septic shock and thiamine deficiency exhibited a significant improvement in the mortality rate after applying intravenous thiamine infusion [13]. Although patients with thiamine deficiency would be better served by thiamine replacement therapy, the diagnosis of thiamine deficiency depends mainly on clinical suspicion and therefore might be difficult, especially on admission. In addition most cardiac beriberi leads to high-output heart failure, while some patients exhibit low cardiac output (shoshin beriberi) [14]. Besides, it is well known that the serum thiamine level does not necessarily reflect thiamine deficiency of the whole body [8]. In this context, thiamine deficiency might be underestimated. Not to mention clinical history or physical findings were important; however, measuring ScvO_2_ would help identify patients with thiamine deficiency especially in shock status. Thus, administration of thiamine could improve the mortality rate among critically ill patients.

## Conclusion

High ScvO_2_ might serve as a predictor of thiamine deficiency in critically ill patients, with loop diuretics possibly leading to further depletion of thiamine.

## Abbreviations

ATP: Adenosine triphosphate
ECMO: Extracorporeal membrane oxygenation
IABP: Intra-aortic balloon pumping
PDH: Pyruvate dehydrogenase
ScvO_2_: Central venous oxygen saturation
TTE: Transthoracic echocardiography

## References

[1] Textoris J, Fouché L, Wiramus S (2011). High central venous oxygen saturation in the latter stages of septic shock is associated with increased mortality. Crit Care.

[2] Balzer F, Sander M, Simon M (2015). High central venous saturation after cardiac surgery is associated with increased organ failure and long-term mortality: an observational cross-sectional study. Crit Care.

[3] Singer M (2014). The role of mitochondrial dysfunction in sepsis-induced multi-organ failure. Virulence.

[4] Majima N, Umegaki O, Soen M (2013). Use of central venous saturation monitoring in a patient with pediatric cardiac beriberi. World J Clin Cases.

[5] Sole MJ, Barr A, Keith ME (2006). The prevalence of thiamin deficiency in hospitalized patients with congestive heart failure. J Am Coll Cardiol.

[6] Sica DA (2007). Loop diuretic therapy, thiamine balance, and heart failure. Pharmacology in Congestive Heart Failure.

[7] Larkin JR, Zhang F, Godfrey L (2012). Glucose-induced down regulation of thiamine transporters in the kidney proximal tubular epithelium produces thiamine insufficiency in diabetes. PLoS One.

[8] Mallat J, Lemyze M, Thevenin D (2016). Do not forget to give thiamine to your septic shock patient!. J Thorac Dis.

[9] Misumida N, Umeda H, Iwase M (2014). Shoshin beriberi induced by long-term administration of diuretics: a case report. Case Reports Cardiol.

[10] Kountchev J, Bijuklic K, Bellmann R (2005). A patient with severe lactic acidosis and rapidly evolving multiple organ failure: a case of shoshin beri-beri. Intensive Care Med.

[11] Diltoer MW, Troubleyn J, Lauwers R (2004). Ketosis and cardiac failure: common signs of a single condition. Eur J Emerg Med.

[12] Ito M, Tanabe Y, Suzuki K (2002). Shoshin beriberi with vasospastic angina pectoris. Circ J.

[13] Donnino MW, Andersen LW, Chase M (2016). Randomized, double-blind, placebo-controlled trial of thiamine as a metabolic resuscitator in septic shock. Crit Care Med.

[14] McIntyre N, Stanley NN (1971). Cardiac beriberi: two modes of presentation. Br Med J.

